# Molecular Dynamics Analysis of Multi-Factor Influences on Structural Defects in Deposited Mg-Matrix Zn Atom Films

**DOI:** 10.3390/ma17194700

**Published:** 2024-09-25

**Authors:** Zhen Zhou, Chaoyue Ji, Dongyang Hou, Shunyong Jiang, Yuhang Ouyang, Fang Dong, Sheng Liu

**Affiliations:** 1 The Institute of Technological Sciences, Wuhan University, Wuhan 430072, China; zhen.z@whu.edu.cn (Z.Z.); 2018106520021@whu.edu.cn (C.J.); 2022106520011@whu.edu.cn (D.H.); 2023206520002@whu.edu.cn (S.J.); 2022206520021@whu.edu.cn (Y.O.); 2 Wuhan Institute of Quantum Technology, Wuhan 430206, China; 3 School of Power and Mechanical Engineering, Wuhan University, Wuhan 430072, China

**Keywords:** molecular dynamic, deposited structural defects, Mg-matrix Zn film

## Abstract

Mg metal vascular stents not only have good mechanical support properties but also can be entirely absorbed by the human body as a trace element beneficial to the human body, but because Mg metal is quickly dissolved and absorbed in the human body, magnesium metal alone cannot be ideally used as a vascular stent. Since the dense oxide Zn film formed by Zn contact with oxygen in the air has good anti-corrosion performance, the Zn nanolayer film deposited on the Mg matrix vascular scaffold by magnetron sputtering can effectively inhibit the rapid dissolution of Mg metal. However, there are few studies on the molecular dynamic structural defects of Mg-matrix Zn atomic magnetron sputtering, and the atomic level simulation of Mg-matrix Zn thin-film depositions can comprehensively understand the atomic level structural defects in the deposition process of Zn thin films from a theoretical perspective to further guide experimental research. Based on this, this research first studied and analyzed the atomic layer structure defects, surface morphology, surface roughness, atomic density of different deposited layers, radial distribution function, and residual stress of the thin-film deposition layer of 1500 deposited Zn atoms at the initial deposition stage, during and after deposition under Mg-matrix thermal layer 500K and a deposited velocity 5 Å/ps by molecular dynamics. At the same time, the effects of temperature and deposited velocity of the Mg-matrix thermal layer on the surface morphology, roughness, and biaxial stress of the deposited films were studied.

## 1. Introduction

The total number of cardiovascular disease (CVD) cases nearly doubled from 271 million in 1990 to 523 million in 2019. Additionally, CVD deaths increased from 12.1 million in 1990 to 18.6 million in 2019 [1,2]. Presently, Percutaneous Coronary Intervention (PCI) stands as one of the foremost effective therapeutic modalities for angiocardiography; within this context, the vascular stent, serving as the principal apparatus, has garnered significant attention across the medical and academic communities [3,4]. Over the past two decades, there has been a significant surge in interest regarding the development of degradable vascular stents, culminating in the progression of both polymeric- and metallic-based designs to clinical trial phases. This burgeoning field underscores a pivotal shift in cardiovascular interventions, aiming to enhance patient outcomes through the utilization of temporary scaffolding that naturally dissolves, thereby reducing long-term complications associated with permanent stent implantation [5]. The burgeoning interest in magnesium (Mg) alloys as a viable option for short-term structural biomaterials, particularly in applications such as orthopedic implants and cardiovascular stents, is primarily attributable to their unique amalgamation of biocompatibility, biodegradability, and tunable mechanical properties. This distinctive blend of characteristics positions magnesium alloys as a promising candidate in the realm of medical device development, offering potential advances in patient care and treatment outcomes [6,7,8]. Magnesium (Mg) has garnered significant attention within biomaterials and biomedical implants, primarily due to its inherent biodegradability and superior mechanical strength when contrasted with polymeric materials. The combination of Mg’s biodegradation properties, biocompatibility, and superior mechanical strength underscores the rationale behind the extensive exploration of Mg as a viable biomaterial [9]. The fourth-generation biodegradable vascular stent, composed of magnesium, possesses the unique ability to be fully absorbed by the human body as an essential trace element while exhibiting no toxic or adverse effects [10,11,12]. The fracture toughness of magnesium surpasses that of ceramic biomaterials, such as hydroxyapatite [12]. The elastic modulus and compressive yield strength of magnesium exhibit closer proximity to those of natural bone in comparison to other commonly utilized metallic implants [13]. Furthermore, magnesium plays a crucial role in human metabolism and is naturally present in bone tissue [14,15]. Additionally, numerous studies have indicated that individuals undergoing dialysis and exhibiting elevated Mg serum levels may experience a degree of protection against vascular calcification [16,17]. The inhibition of crystal growth may represent a plausible mechanism underlying its protective efficacy [18].

However, pure magnesium exhibits deficiencies in both corrosion resistance and mechanical strength [19,20,21,22,23]. Generally, in the realm of corrosion resistance enhancement, two primary approaches have garnered widespread adoption: element alloying and surface treatments [15,19]. Recent research has predominantly emphasized the biochemical aspect of a two-step alkali-fluoride treatment applied to magnesium alloy [24,25,26]. This treatment is effective in eliminating the second phase from the substrate surface and in creating a dense, impeccable magnesium fluoride (MgF2) coating [24]. Consequently, it bestows superior corrosion resistance upon the magnesium alloy [26]. In addition, adding Al, In, Mn, Zn, or Zr reduced the corrosion rate of as-cast Mg–X alloys in both SBF and Hank’s solutions [27]. The cytotoxicity tests indicated that Mg–1Al, Mg–1Sn, and Mg–1Zn alloy extracts showed no significant reduced cell viability to fibroblasts (L-929 and NIH3T3); Mg–1Al, Mg–1Si, Mg–1Sn, Mg–1Y, Mg–1Zn, and Mg–1Zr alloy extracts indicated no significant toxicity to osteoblasts (MC3T3-E1); and Mg–1Al and Mg–1Zn indicated no adverse effect on viabilities of the blood-vessel-related cells ECV304 and VSMC. In the hemolysis tests, Mg–1In, Mg–1Mn, Mg–1Si, and Mg–1Y alloys induced less than 5% hemolysis [27]. Zinc coatings have been widely used to extend the lifetime of steel products for biomedical devices, automobiles, home appliances, and electronics due to their inherent corrosion resistance [28,29,30]. The deposition of Mg-matrix Zn films solves the problem of the easy corrosion of Mg vascular stents and improves the mechanical properties of radial expansion of vascular stents. Further investigation is needed to reduce defects and improve the quality of the Mg matrix Zn coating film. A deeper understanding of the growth mechanism and microstructure at the nanoscale is essential, along with simulation studies of the fundamental theory. Moreover, there are relatively few studies on the surface modification of the Mg matrix by deposition technology, and there are few reports on the preparation of biodegradable metal films [31]. Meanwhile, there are a few studies on the growth structural defects of Mg-matrix Zn film and the complex deposition processes of Mg-matrix Zn atomic magnetron sputtering using molecular dynamics. For example, some studies have found that in the relationship between film defects and corrosion, the coating thickness, defects, porosity, and corrosion through the holes of the coating increase, which aids in the corrosion process and reduces the corrosion resistance of the coating [32]. Some studies have found in the relationship between density and film defects that the coating with dense crystal can fill intrinsic defects of the substrate and provide favorable conditions for obtaining compact passivation film, and the better corrosion resistance was attributed to the fewer surface defects and high electric resistance [33]. An atomic level simulation of Mg-matrix Zn thin-film deposition can comprehensively understand the atomic level structural defects in the deposition process of Zn thin films from a theoretical perspective to further guide experimental research [34].

In order to clearly understand the deposition mechanism of thin films and the influence of various deposition parameters on the deposition of thin films at the atomic scale, this paper establishes the substrate model of Mg atoms in different functional layers by the molecular dynamics method, considers different deposition parameters in turn, and simulates the deposition process of Zn atoms on the Mg atomic substrate of different functional layers under different deposition parameters. Meanwhile, this study examines the evolution of surface defects and mechanical properties of Zn nano-thin-film coatings deposited by Mg alloy vascular scaffolds. Using molecular dynamics, the study investigated the influence of surface forming defects and mechanical properties of Zn nano-thin-film coatings with varying incident velocities and temperatures of the substrate formed by metal Mg substrate deposition of Zn-atom nano-thin-film coatings. The atomic layer structure defects, surface morphology, surface roughness, atomic density of different deposited layers, radial distribution function, and residual stress of the thin-film deposition layer were studied and analyzed.

## 2. Modeling and Simulation Methods

### 2.1. Simulation Model

**Figure 1:**
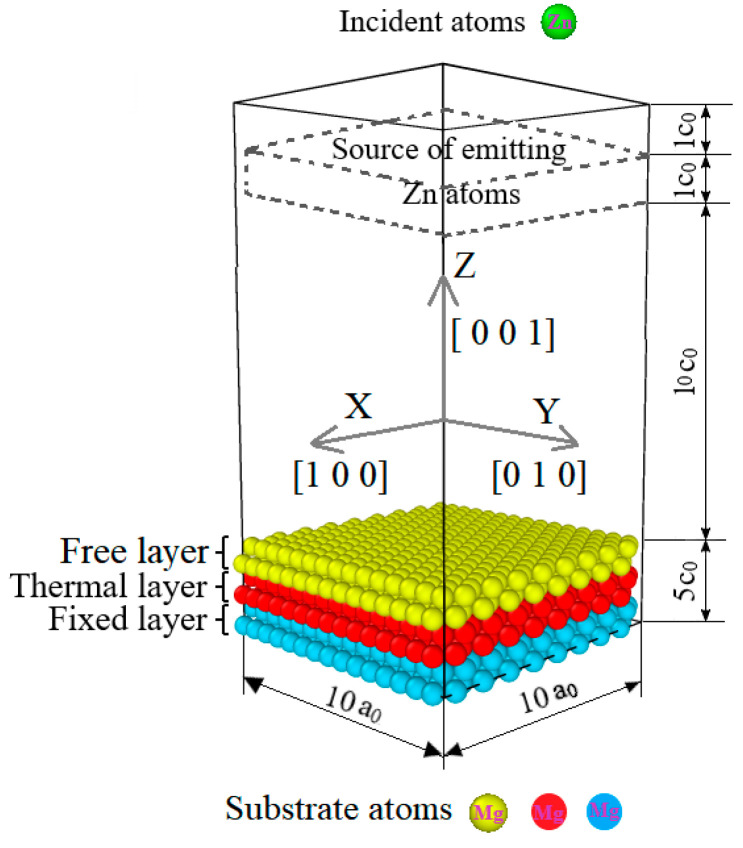
Mg-matrix Zn deposition model.

The dimension of the substrate was 4.7 × 4.7 × 1.5 nm, divided into three parts. The first part is a fixed part with the size of 4.7 × 4.7 × 1.5 nm, which controls the movement of the incident atoms. The second part is the thermal control part with a 4.7 × 4.7 × 0.5 nm size, which controls the initial temperature with the canonical ensemble. The third part is the free part with a size of 4.7 × 4.7 × 0.5 nm, which absorbs the kinetic energy of the incident atoms with the micro-canonical ensemble [35]. The deposit direction is along the [0 0 1] (z-axis) orientation and perpendicular to the [0 0 1] direction of the Mg atom with a total of 2500 atoms—the simulation model is shown in Figure 1.

### 2.2. Simulation Method

Selecting appropriate potential functions can more accurately reflect the different action properties for the interaction between different atoms. This article uses the optimized embedded-atom-method interatomic potential [36]; the potential function has been successfully applied to study the interaction properties of different Mg–Zn systems [37,38]. Therefore, the modified embedded-atom-method interatomic potential was chosen to depict the interatomic potential of the Mg–Zn IMC in this work.

In the simulation, the Mg atom was deposited onto the substrate surface with the timestep of 1 fs (1 × 10^−15^ s) and the accident velocity of 0.1, 0.5, 1, 1.5, and 2 nm/ps. To avoid insufficient sputtering power density, the substrate kept a temperature high enough to have inter-diffusion in the growing film to form a uniform phase [39]. For this reason, several researchers have elected to deposit pure Mg or Zn–Mg alloy coatings on Zn-plated steel, followed by heat treatment at temperatures above 300K to obtain Zn–Mg intermetallic phases [34,39]. In this simulation, the total deposition time was 100 ns. The deposition velocity was 5 Å/ps, and the temperature of the thermal control layer varied from 300 K, 500 K, 700 K, to 900 K. The initial temperature of the thermal control layer was based on the solubility of Mg atoms [40]. The statistical analysis of the atomic number, the coverage of the deposition atoms, and radial distribution function (RDF) were performed to investigate the deposition of Zn films through varying incident velocities and substrate temperatures. Molecular dynamics simulations were performed using Lammps stable version 2022 software [41,42], and the results were visualized using Ovito 3.10.6 visualization software [43].

## 3. Results and Discussion

### 3.1. Crystalline Structure, Surface Morphology, and Roughness of the Deposited Film

**Figure 2:**
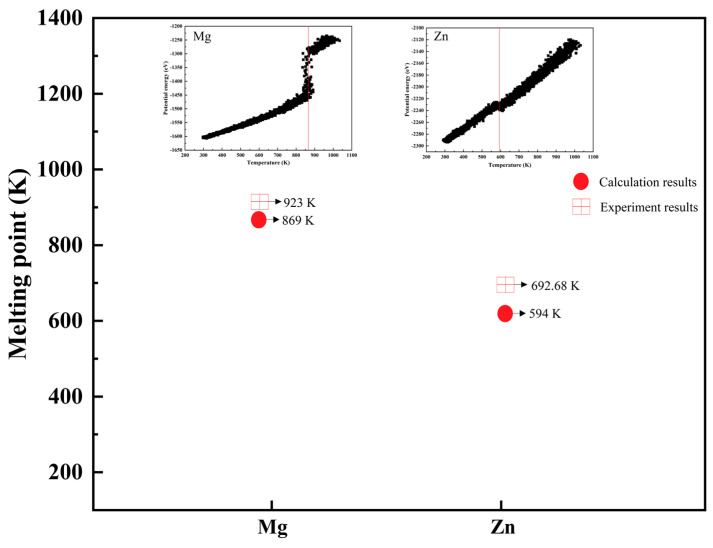
The curve of potential energy and heating temperature.

**Figure 3:**
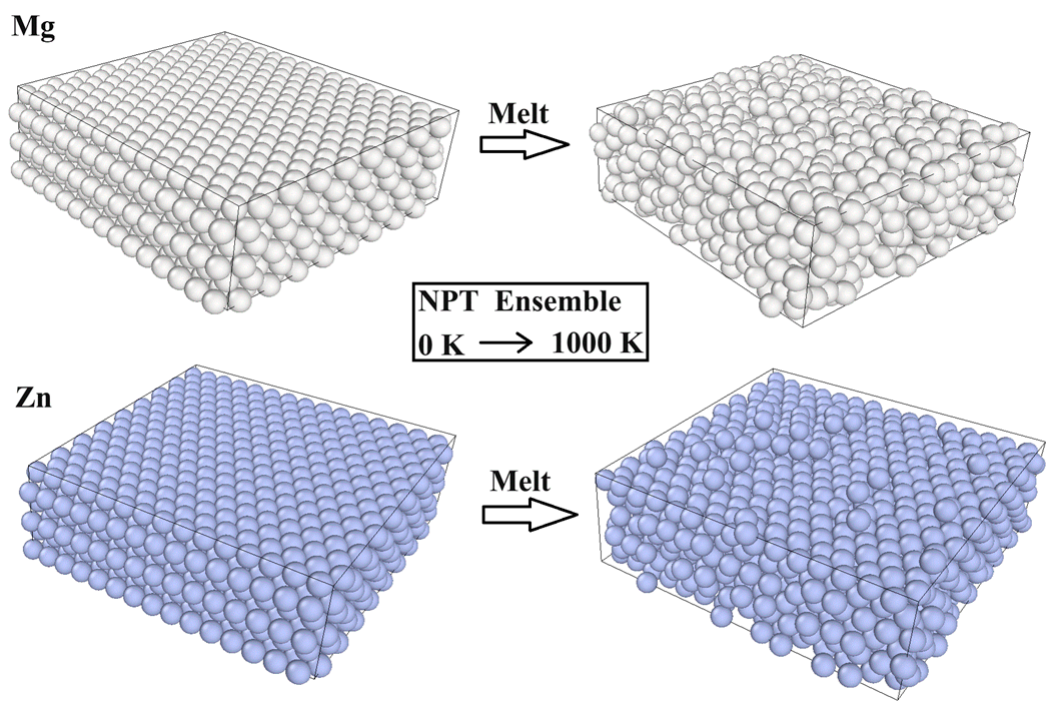
The molecule dynamic for melting results in Mg and Zn.

To verify the accuracy of the simulation system, we calculated the melting points of Mg and Zn using a modified embedded-atom-method interatomic potential. The Zn and Mg bulk models consisted of approximately 1530 atoms, respectively. The system was relaxed with the NPT ensemble, and the timestep was 50 ps. The NPT ensemble was used to heat the systems continuously from room temperature to 1000K, and the timestep was 200 ps. The curves of potential energy and heating temperature are shown in Figure 2, and the molecule dynamics for melting resulting in Mg and Zn are shown in Figure 3. The overheating ratios of the calculated melting points of Zn and Mg were −14.2% and −5.8%, respectively. Due to the influence of the number of atoms in the model system and the thermal effect during the simulation, the experimental value of the atomic melting point deviated from the simulation results, similar to those reported in several studies [35,44,45,46]. This indicates that the simulation method utilized in this study holds significance.

**Figure 4:**
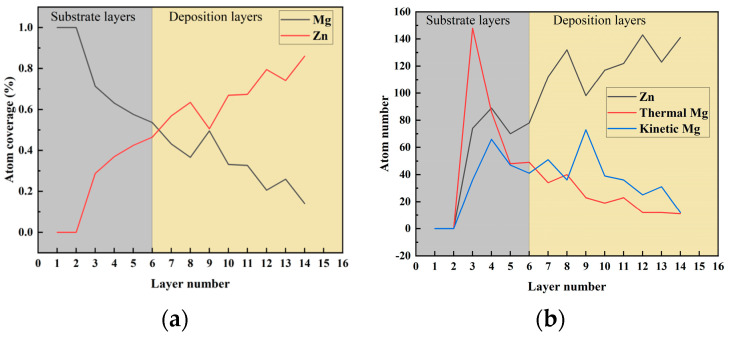
Different atom coverage for different layers at a thermal part temperature of 500 K and the deposited velocity of 5 Å/ps. (**a**) The content for Mg and Zn coverage for various layers. (**b**) Atoms of different functional layers covered for other layers.

In order to study the coverage of the layer containing the Mg–Zn compound during the deposition of Zn atoms, we used the *z*-axis of the Mg substrate as the reference point. The coverage was calculated as the ratio of the counted atoms to the number of atoms in a complete layer of the Mg substrate, which consists of 255 atoms. In order to study the process of deposition defects, the thermal part temperature of 500 K and the deposited velocity of 5 Å/ps were used as the specific metrics. We chose specific metrics, such as 5 Å/ps and 500 K, because some studies have found that to be successful at low substrate temperatures during deposition, atoms may not have sufficient energy to diffuse to regions with lower energy, resulting in more considerable atomic stresses in the deposited structures [47,48]. The atomic coverage of Zn atoms deposited on the Mg substrate at a thermal part temperature of 500 K and the deposited velocity of 5 Å/ps is shown in Figure 4.

It can be seen from Figure 4a that the deposition Zn atoms entered the basal Mg atomic layer. With the gradual increase in the deposition layer, the coverage of Mg atoms gradually decreased, and the coverage of Zn atoms gradually increased. The Zn atom entered the third layer of the substrate, the Thermal Mg layer. It can be seen from Figure 4b that during the deposition of Zn atoms, the coverage of Kinetic layer Mg atoms and Thermal layer Mg ones decreased with the gradual increase in the deposition layer. Among them, the number of Thermal layer Mg atoms in the fifth to sixth layers of the interface layer was more than the number of Kinetic layer Mg ones, which was mainly due to the thermal motion, and the thermal layer Mg promoted the movement of a large number of Mg atoms to the free Kinetic Mg layer. When the number of atomic layers exceeded the ninth layer, the number of Kinetic Mg atoms was greater than that of Thermal Mg ones as the number of nuclear layers increased.

**Figure 5:**
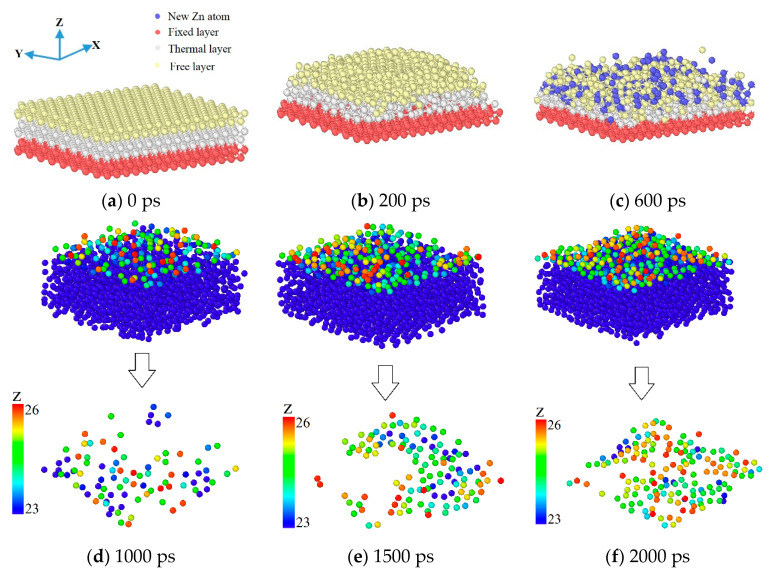
Time-dependent snapshots of Zn deposited on Mg substrate at a thermal part temperature of 500 K and a deposited velocity of 5 Å/ps.

The deposition process of Zn atoms was determined by studying the effect of the deposition of Zn atoms on the Mg substrate. As shown in the figure above, Figure 5a–c are distinguished by different colors to represent the newly deposited Zn atoms. Figure 5a shows the initial stage; the deposition had not yet begun. When the time reached 200 ps, it can be seen from Figure 5b that the Mg base surface atoms started to melt from the periphery, and when the time reached 600 ps, it can be seen that the Zn atoms on the surface were gradually deposited. The depth of new Zn atom deposition was the deepest around the basement, mainly because the melting started from the periphery of the substrate in Figure 5b, and the Zn atom deposition effect of the new deposition was the best. Figure 5d–f show the schematic results of the deposition of the most superficial Zn atoms at different deposition times at a thermal part temperature of 500 K and a deposited velocity of 5 Å/ps. In addition, the surface of the deposition of thin films was formed in the form of islands through the combination of Mg atoms and Zn atoms and the combination of Zn atoms and Zn atoms.

The defects in the interfacial layer Zn atom deposition were investigated further to study the vacancy defects in the deposition process.

**Figure 6:**
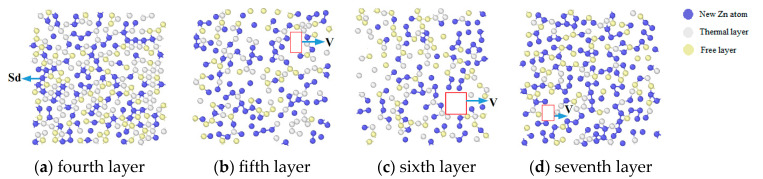
Atomic layer structure defects for different layers of the Mg matrix.

From Figure 6a–d, it can be seen that the sixth layer of the Mg matrix had a prominent vacancy cluster, and compared with the other layers, the most superficial layer of the Mg matrix had more vacancy clusters. Figure 6a had more vacancies (Vs) and substitutional dopants (Sds) because the surface Mg atoms were detached from their original positions due to the impact of the deposited Zn atoms with a significant impulse. When the sedimentary layer was closer to the surface layer of the Mg matrix interface, the middle layer in Figure 6b gradually transformed into a vacancy cluster. In the sixth layer, that is, the surface layer of the Mg matrix, the number of vacancies was the largest because the surface layer of the interface layer, the Mg matrix, was most seriously impacted by the deposited Zn atoms. Many Mg vacancies caused by the impact of Zn atoms formed a vacancy group. In layer 7 of Figure 6d, the number of vacancies decreased due to the increase in the number of Zn atoms in the sedimentary layer, which occupied the vacancies.

**Figure 7:**
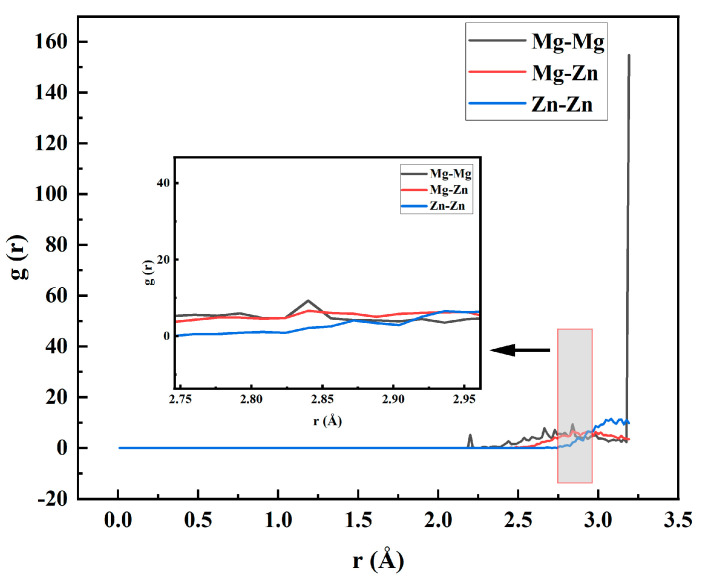
Radial distribution function (RDF) of deposited layers with Zn under 500 K.

The RDF of the deposited Zn atoms in the surface layer of the Mg matrix was further analyzed in Figure 7, and the changes in the structural densities of Mg and Zn atoms were analyzed. When r(Å) >= 2.856 Å, the density distribution probability between Mg–Zn and Zn–Zn was always greater than that between Mg–Zn and Zn–Zn, and with the gradual deposition of Zn atoms, when r (<Å) = 2.856 Å, the density distribution probability between Mg–Zn and Zn–Zn gradually exceeded that of Mg–Mg. This meant that the Zn atoms gradually bound to the Mg atoms and were gradually deposited and entered the Mg basal layer when r (Å) > 2.936 Å. The density distribution probability between Zn–Zn and Zn–Zn was more significant than that between Mg–Zn and Zn–Zn, which meant that when the mixed layer of the Zn and Mg atom interface was formed, with the gradual deposition of Zn atoms, Zn atoms and Zn atoms combined to create a Pure Zn atomic deposition layer.

**Figure 8:**
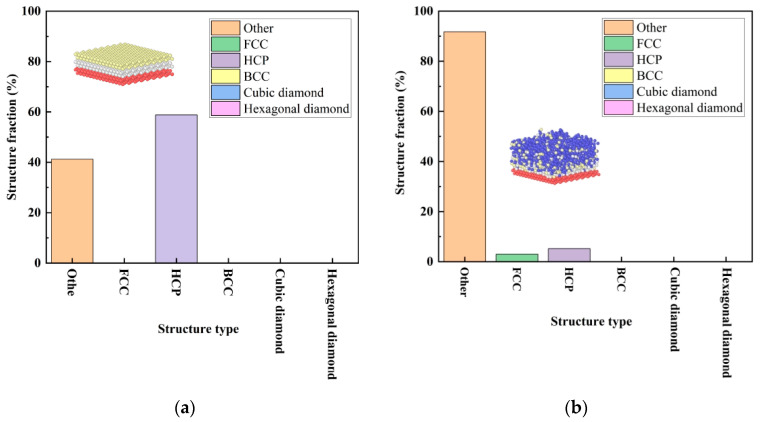
Various structure fractions at a thermal part temperature of 500 K and a deposited velocity of 5 Å/ps. (**a**) The beginning of the deposition. (**b**) The end of the deposition.

The change in crystal structure can be reflected. At a thermal part temperature of 500 K and a deposited velocity of 5 Å/ps, it can be seen from the different crystal structure diagrams from the beginning to the end of the deposition that the crystal structure in Figure 8a is mainly a 58.8% HCP structure and a 41.2% other crystal structure. The crystal structure in Figure 8b is mainly 91.7% other crystals, a small amount of 5.2% HCP crystals, and a small amount of 3.0% FCC crystals.

**Figure 9:**
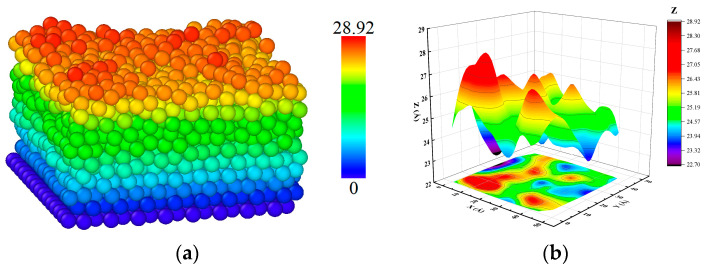
Surface morphology at a thermal part temperature of 500 K and a deposited velocity of 5 Å/ps. (**a**) Atomic morphology. (**b**) Surface morphology of the deposited film.

The deposition of the deposited film at a temperature of 500 K was outputted using visualization software, as shown in Figure 9a. The surface morphology was plotted with the surface atomic coordinates of the deposited film at a temperature of 500 K, as shown in Figure 9b. The final result of the deposited surface morphology is shown in Figure 9b. The morphological analysis showed that the deposition surface was rough due to the fluctuations of atoms received from the upper surface film at a thermal part temperature of 500 K and a deposited velocity of 5 Å/ps, simultaneously releasing the internal stress between the sedimentary layers and form an island-like structure as seen in Figure 9.

The roughness calculation formula further calculated the roughness of the deposited surface at a temperature of 500K.

$$R_{a}=\sqrt{\frac{\sum_{1}^{N}{\left(Z_{i}-\bar{Z}\right)}^{2}}{N}}$$

The coordinates of the deposited surface of atom *Z_i_* denote the *Z* direction, $\bar{Z}$ denotes the average height of all surface atoms in the *Z* direction, and the N table represents the number of surface atoms. The final surface roughness at a 500 K substrate deposition temperature was calculated to be 1.21 Å. As can be seen from the atomic roughness diagram (b), the edge of the sedimentary surface was rough due to the form of islanding nucleation.

### 3.2. The Residual Stress and Atomic Layer Density of the Deposited Film

Since the internal stress of the deposited film is the critical factor leading to the quality of the deposition of the film, the evolution of the residual stress during the deposition process was characterized by the average mean biaxial stress during the deposition process to analyze the influence of the residual stress during the deposition process on the deposition quality of the deposited film.

$$\sigma_{p}^{avg}={\left(\sigma_{xx}^{avg}+\sigma_{\mathrm{yy}}^{avg}\right)}^{2}$$

The average biaxial stress and the average atomic stress for atoms in layers X and Y correspond to the exponents in the stress tensor, with “p” representing the number of atoms within the layer.

**Figure 10:**
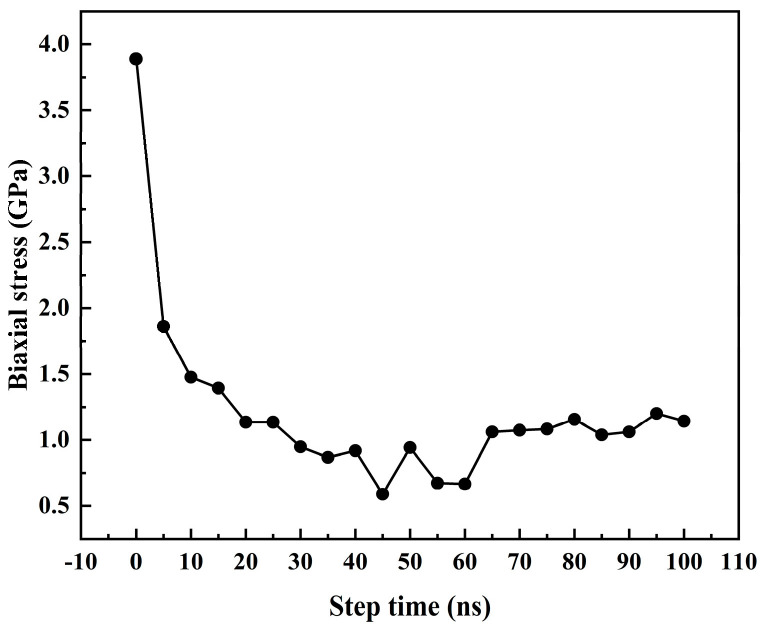
The average biaxial stress with the increase in step time at a thermal part temperature of 500 K and a deposited velocity of 5 Å/ps.

From the analysis in the Figure 10, the biaxial stress gradually decreased when the step time was from 0 ns to 60 ns, and the minimum biaxial stress was 0.6583 GPa when the step was 60 ns. In the step time from 0 to 10 ns, the initial biaxial stress of Zn atom deposition gradually decreased due to the atoms’ gradual melting in the Mg-matrix surface layer. The internal tension of the deposition system should be more significant in the initial stage of melting, and the overall tensile stress of the deposition system was affected by thermal expansion. The internal stress gradually decreased with the gradual dissolution of the surface. The compressive stress formed by Zn atoms during the deposition process of Zn atoms in the deposition process canceled out part of the tensile stress. When the step time was 45 ns, the minimum tensile stress was 0.58986 GPa. With the gradual completion of Zn atomic deposition, the deposition atoms were squeezed against each other in the finite volume within the system, resulting in a slight increase in the internal stress. After the atomic deposition, the system’s biaxial stress was relaxed, and the step time exceeded 65 ns. However, when the value of the biaxial stress of the entire system was in a state of oscillation, the amplitude of the value was relatively small, and the biaxial stress inside the system remained relatively stable, in which the internal interaction between consecutive layers may have caused the resulting stress value to exhibit a similar trend as that of Zientarski and Zhou et al. [49,50].

**Figure 11:**
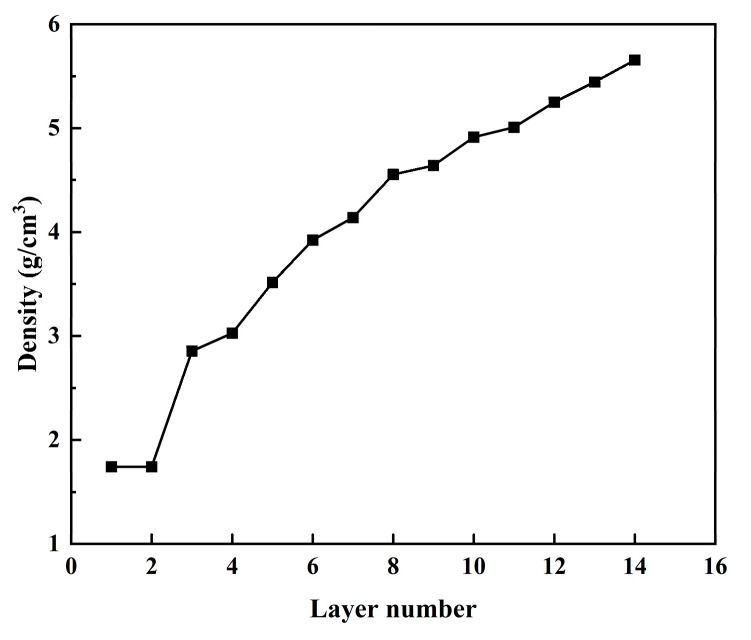
The atomic layer density of the deposited film with different layer numbers at a thermal part temperature of 500 K and a deposited velocity of 5 Å/ps.

Because the quality of the atomic layer deposition film is related to the density of each layer, it was necessary to calculate the thickness of each layer of the final structure of Mg-matrix Zn atomic deposition. The density of mass was obtained by the following:

$$\rho =\frac{m_{Zn}+m_{Mg}}{V_{Zn}+V_{Mg}}$$

where $m_{Mg}$ is the mass of Mg atoms in the layer and $m_{Zn}$ is the mass of Zn atoms in the layer. $V_{Zn}$ is the volume of Zn atoms in the layer and $V_{Mg}$ the volume of Mg atoms in the layer. The density of the deposited different layer at a thermal part temperature of 500 K and the deposited velocity of 5 Å/ps are shown in Figure 11.

The higher the density between the deposited layers of the deposited film, the better the thickness of the deposited film [51]. It can be seen from the above figure that with the gradual increase in the deposited layer, the density of the deposited film gradually increased from 1.74 g/cm^3^ to 5.65 g/cm^3^ of the base layer, which was between Mg and Zn (1.74 g/cm^3^ and 7.14 g/cm^3^, respectively).

### 3.3. Effect of the Different Thermal Temperatures

**Figure 12:**
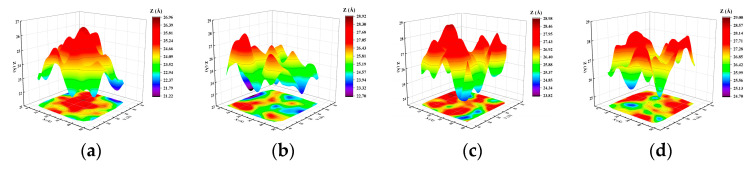

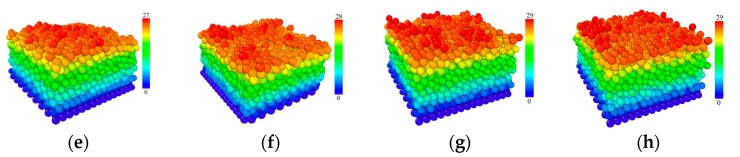
Atomic morphology (**a**–**d**) and surface morphology (**e**–**h**) of the deposited film at different thermal temperatures and a deposited velocity of 5 Å/ps: (**a**) and (**e**) at 300 K; (**b**) and (**f**) at 500 K; (**c**) and (**g**) at 700 K; (**d**) and (**h**) 900 K.

It can be seen from Figure 12a–d above that when the number of Zn atoms deposited was constant, the height of the sedimentary layer of atomic morphology increased gradually with the gradual growth of the thermal temperature of the Mg substrate. The final sedimentary surface morphology diagram of Figure 12a corresponds to the surface morphology of Figure 12e, and the final sedimentary topography shows that most of the sedimentary locations are concentrated in the middle. With the increase in the Mg substrate’s thermal temperature, the Zn atomic deposition area from the atomic morphology diagram in Figure 12a–d and the surface morphology diagram changed from the middle part to a three-dimensional island structure pattern and gradually turned to the peripheral edge. The main reason was that when the sedimentary layer’s temperature was low, the substrate Mg atoms did not melt at room temperature, and the fluctuations between atoms were small. When the thermal temperature of the substrate gradually increased, the Mg substrate melted. From Figure 12a–c of the time-dependent snapshots of Zn deposited on the Mg substrate, it can be seen that the dissolution of substrate Mg started from the surrounding edges of the substrate. When the melting temperature gradually increased to 900 K, the atoms around the edge of the Mg substrate were the most active, and the final result of the Zn atomic deposition showed that the three-dimensional island structure of the surface morphology was the highest at the edge of the surrounding edge.

**Figure 13:**
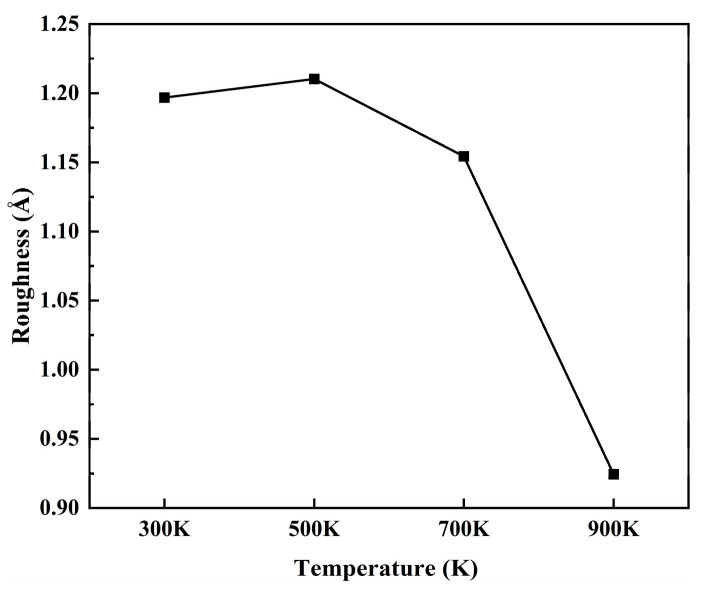
The surface roughness of deposited film at different temperatures.

As seen in Figure 13 above, the surface roughness of the deposited film gradually decreased as the temperature of the thermal layer gradually increased. When the temperature of the thermal layer was 900 K, the surface roughness of the thin-film deposition was 0.92441 Å. The rise in base temperature enhanced the deposition of thin films, resulting in a finer surface morphology. Improved surface morphology, in turn, helped enhance the resistance of Mg-matrix Zn thin film vascular stents to external corrosion.

**Figure 14:**
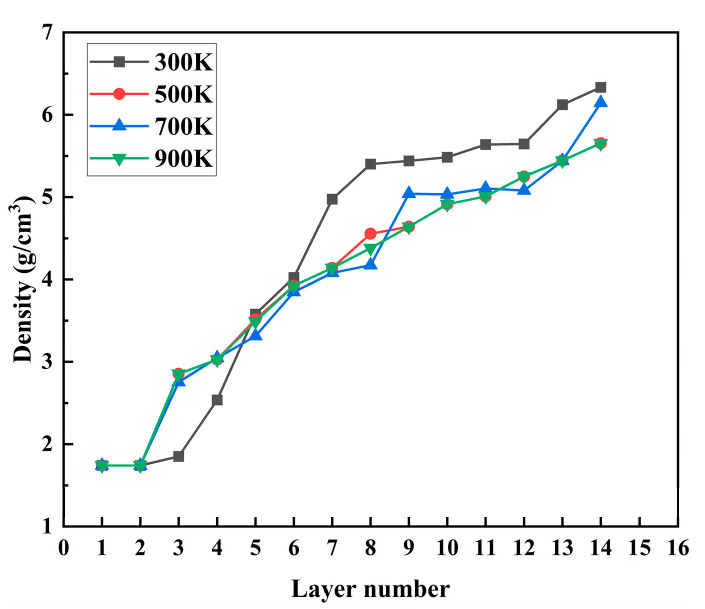
The atomic layer density of the deposited film at different thermal temperatures and the deposited velocity of 5 Å/ps.

As seen from Figure 14 above, from layer 0 to layer 5, with the increase in temperature, the density of the Mg basis at 300 K was lower than that at other temperatures. With the temperature rise, the fifth and sixth layers became the interface layers. After the interface layer, the thermal temperature at 300 K was higher than the density at other temperatures. This was mainly because the Mg group did not melt at 300 K. At room temperature, the Zn atoms were deposited on the Mg group, and the Mg atoms on the interface were not fully expanded into the deposited Zn thin-film layer. The Mg base layer gradually melted with the increase in the thermal temperature layer. With the deposition, the Mg atoms diffused progressively into the Zn thin-film deposition layer. When the Zn atom deposition layer exceeded the interface layer, the density of the deposition layer at 500 K, 700 K, and 900 K was lower than that at 300 K at room temperature. It can be seen that the increase in substrate temperature was not sensitive to the change in crystal density.

From the beginning of Figure 15①, the internal stress of the Mg matrix in the dissolved state was more significant. The inter-atomic internal stress gradually decreased during the gradual dissolution process. In Figure 15②, the Zn atoms were gradually deposed. With the continuous deposition of Zn atoms, the aggregation density of the thin film increased, which provided specific compressive stress, which gradually increased with the deposition of Zn atoms and offset a part of the tensile stresses, as can be seen from the figure. When the step time gradually increased to 60 ns, the tensile stress of the system gradually decreased during the deposition of the thin film. When the step time gradually increased from 60 ns to 80 ns, the amount of Zn atoms deposited was limited, and the deposition was slowly completed. The interaction in the finite volume led to the gradual increase in internal stress, and the Zn atoms in Figure 15③ were in a relaxed state after deposition. The whole system was relatively stable, and the biaxial stress inside the system remained relatively stable. It can be seen that with the increase in temperature, the residual stress of the final deposited film gradually decreased.

From the analysis of the surface morphology, roughness, internal stress, and crystal density of the film at different thermal temperatures of the substrate, it can be seen that the increase in the substrate temperature was more conducive to the formation of Mg-based Zn films. When the surface roughness of the film was more minor, the surface morphology was finer, and the internal stress of the film was smaller. Finally, it is more conducive to improving the corrosion resistance of Mg-based Zn thin-film vascular scaffolds in human blood.

### 3.4. Effect of the Different Deposited Velocity

**Figure 16:**
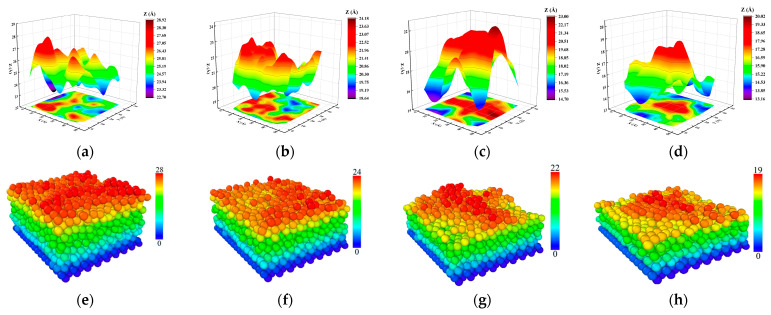
Atomic morphology (**a**–**d**) and surface morphology (**e**–**h**) of the deposited film at different deposited velocities: (**a**) and (**e**) at 5 Å/ps; (**b**) and (**f**) at 10 Å/ps; (**c**) and (**g**) at 15 Å/ps; (**d**) and (**h**) 20 Å/ps.

Regarding atomic morphology, from Figure 16, (a–d), it can be seen that the height of atomic morphology gradually decreases. Regarding surface morphology (e–h), it can be seen that with the gradual increase in velocity, the three-dimensional island structure formed by the final deposition of the surface morphology was distributed around (e) and (f) and gradually concentrated in the middle, such as (g) and (h), with the increase in velocity. As the deposition speed increased, the thickness of the deposited film decreased. It can be concluded that the significant deposition rate was not conducive to the deposition of Mg-based Zn thin films, nor was it conducive to the deposition of Mg-matrix Zn thin film vascular stents.

**Figure 17:**
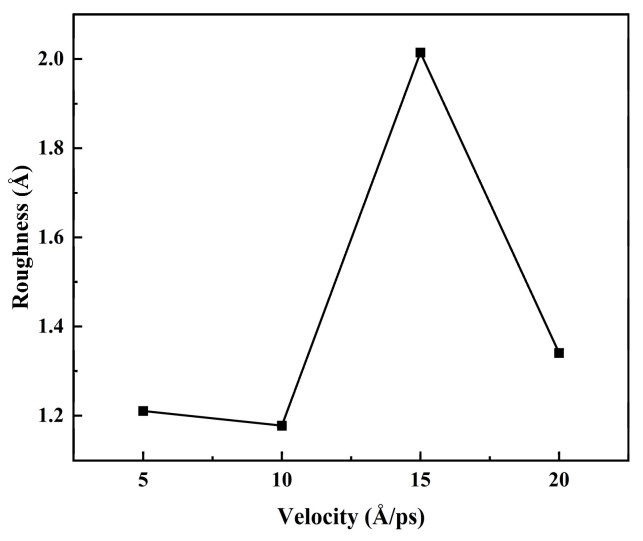
The surface roughness of deposited film at different velocities.

In Figure 17, from the surface roughness of the deposited film at different speeds, when V = 10 Å/ps, the surface roughness of the deposited film was the smallest and the surface roughness of the deposited film is 1.17746 Å. When V = 15 Å/ps, the surface roughness of the deposited film was the maximum at 2.01447 Å. When V = 20 Å/ps, the surface roughness of the deposited film was 1.34057 Å, and the difference in surface roughness was more significant. It was observed that the surface morphology and roughness were closely related to the deposited velocity.

**Figure 18:**
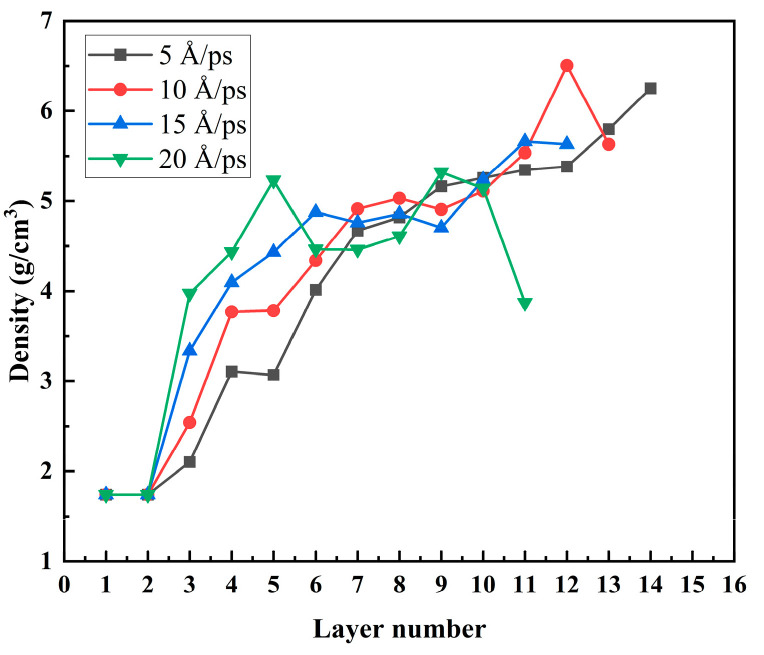
The atomic layer density of the deposited film with different layer numbers under different velocities under a temperature of 500 K.

As seen from the Figure 18, from layer 0 to layer 5, the deposited velocity had a lower density of Mg-base at V = 5 Å/ps than at other temperatures as the temperature increased. After passing the sixth layer of the interface layer, the density of the final deposition layer gradually decreased with the temperature rise, and the thickness of the film was less than one atomic layer for every 5 Å/ps increase in the deposition velocity. It can be seen that the deposition rate had a significant influence on the density of Mg-matrix Zn atomic films.

From the beginning of the curves, which correspond to the atomic details in Figure 19①, the internal stress of the Mg group in the dissolved state was more significant. The interatomic internal stress gradually decreased during the gradual dissolution process, which corresponded to the atomic details in Figure 19②, and the Zn atoms were gradually deposed. With the continuous deposition of Zn atoms, the aggregation density of the thin film increased, which provided a specific compressive stress, which gradually increased with the deposition of Zn atoms and offset part of the tensile stress. In the Figure 19② stage, with the increase in the Zn atom deposition velocity, the aggregation density of the film increased, which led to the decrease in the tensile stress of the film. At the same time, the velocity of Zn atom deposition impact was more extensive. The momentum generated was more significant when V was less than 20 Å/ps; the overall compressive stress caused to the system was still less than the system’s tensile stress, so the system’s overall stress state was still tensile. When V = 20 Å/ps, the overall compressive stress caused by the deposition of Zn atoms was greater than the tensile stress of the system, and the overall stress state of the system was gradually transformed into compressive stress, which gradually increased with the deposition of Zn atoms. When the deposition velocity reached V = 20 Å/ps, the biaxial stress changed from positive to negative and gradually increased, and the maximum negative biaxial stress was −0.58894 GPa. When V = 20 Å/ps, the step time was from 55 ns to 65 ns, and the biaxial stress changed from negative to positive, the compressive stress gradually decreased and gradually converted into tensile stress, and the tensile stress gradually increased. This was because the amount of Zn atoms deposited was limited, the deposition was gradually completed, and the tensile stress in the finite volume gradually increased. The Zn atom was relaxed after deposition, the whole system was relatively stable, and the biaxial stress remained relatively stable, corresponding to the atomic details in Figure 19③. Since the biaxial stress represents the residual stress in the system, the overall trend of the residual stress of the system was consistent with the increase in step time under different temperatures of the Mg-matrix thermal layer and different deposited velocities of the Zn atom.

According to the analysis of the surface morphology, roughness, internal stress, and crystal density of the film at different deposition speeds of the substrate, it can be seen that the surface morphology, roughness, and crystal density were very sensitive to the deposition rate. With the increase in deposition speed, the thickness of the film decreased, and the roughness tended to increase. In addition, with the increase in deposition speed, the internal stress of the final deposited film tended to increase gradually. The rough surface morphology, thinner film, and significant internal stress brought about by the higher deposition rate were not conducive to the formation of Mg-based Zn films and were also highly unfavorable to improving the corrosion resistance of Mg-based Zn thin-film vascular stents in human blood.

## 4. Conclusions

Through the analysis of the atomic layer structure defects, surface morphology, surface roughness, atomic density of different depositary layers, radial distribution function, and residual stress of thin-film deposition layers at the initial stage of Mg-matrix Zn atom deposition, during and after deposition, the analysis of the deposition process of Mg-matrix Zn films showed that Zn atoms were deposited as three-dimensional islands in the Mg matrix. The main reason for the formation of film defects was the presence of vacancies and vacancy clusters in the atomic layer. The increase in the formation of vacancies and vacancy clusters means that Mg-matrix Zn film vascular stents are susceptible to corrosion, which was confirmed given the increase in coating thickness, the defects, porosity, and corrosion through the holes of the coating, which aided in the corrosion process and reduced the corrosion resistance of the coating [32]. Due to the Mg-based thermal layer, the Mg atoms of the substrate were diffused to the coating at the initial stage of the deposition of Zn atoms. The Zn atoms of the coating were also filled to the substrate so that the Zn–Mg compound was formed at the interface coating, which was also proved by the analysis of the radial distribution function during the deposition process. The formation of Zn–Mg compounds led to a faster density growth rate at the interface at the initial deposition stage, which confirmed that the coating with dense crystal could fill intrinsic defects of the substrate and provide favorable conditions for getting a compact passivation film [33]. The formation of Zn–Mg compounds at the interface promoted the increase in crystal density at the interface, which undoubtedly increased the corrosion resistance of Mg-based Zn-coated vascular stents. Biaxial stress was used to characterize the residual stress of different sedimentary layers; the residual stress was 3.88886 GPa at the initial stage of Mg-matrix dissolution, and the overall residual stress was 1.25908 GPa after the whole system was stabilized. The sizeable residual stress inside the film will undoubtedly cause the film to warp, crack, and even fall off, which will cause more severe consequences for Mg-based Zn thin-film vascular stents in radial expansion.

In addition, this paper focuses on the effects of the Mg matrix thermal layer on the surface morphology, atomic density, roughness, and biaxial stress of the deposited layer at different temperatures of 300 K, 500 K, 700 K, and 900 K. The effects of the deposited velocity of Zn atoms on the surface morphology, atomic density, roughness, and biaxial stress of the deposited layer at different deposition velocities were 5 Å/ps, 10 Å/ps, 15 Å/ps, and 20 Å/ps. Through computational studies, it was found that the increase in the substrate temperature was more conducive to the formation of Mg-based Zn films. The surface roughness of the film was more minor, the surface morphology was finer, and the internal stress of the film was smaller. Finally, it was more conducive to improving the corrosion resistance of Mg-based Zn thin-film vascular scaffolds in human blood. According to the analysis of the surface morphology, roughness, internal stress, and crystal density of the film at different deposition speeds of the substrate, it can be seen that the surface morphology, roughness, and crystal density were very sensitive to the deposition rate. With the increase in deposition speed, the thickness of the film decreased, and the roughness tended to increase. In addition, with the increase in deposition speed, the internal stress of the final deposited film tended to increase gradually. The rough surface morphology, thinner film, and significant internal stress brought about by the higher deposition rate were not conducive to the formation of Mg-based Zn films and were also highly unfavorable to improving the corrosion resistance of Mg-based Zn thin-film vascular stents in human blood. The lower deposition rate and higher substrate thermal temperature were more conducive to forming better quality Mg-matrix Zn films.

## Figures and Tables

**Figure 15:**
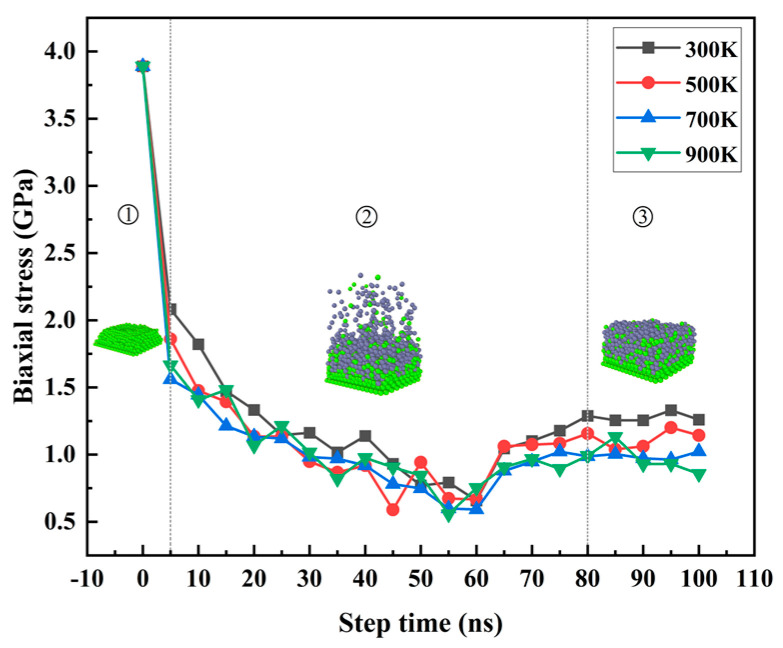
The average biaxial stress with the different step time.

**Figure 19:**
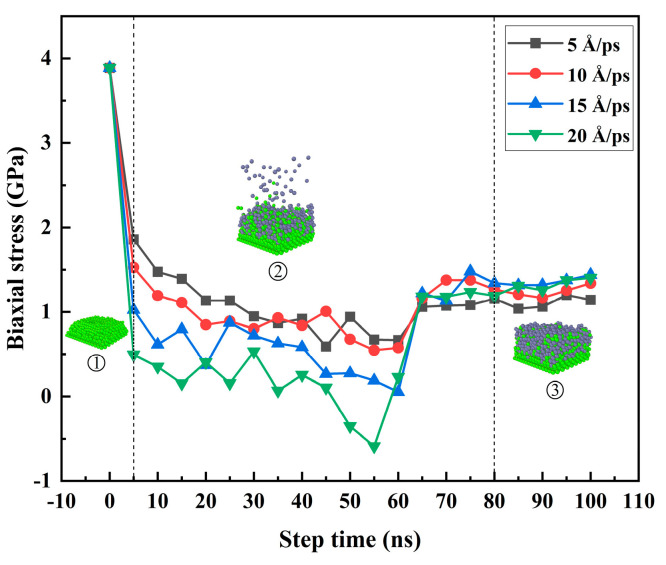
Relationship curves of biaxial stress and step times at different deposited velocities under a temperature of 500K.

## Data Availability

The data presented in this study are openly available in [Mg-Zn atom method interatomic potential] at [http://doi.org/10.1016/j.calphad.2018.01.003] [29].
